# Spatial Characteristics and Regional Transmission Analysis of PM_2.5_ Pollution in Northeast China, 2016–2020

**DOI:** 10.3390/ijerph182312483

**Published:** 2021-11-26

**Authors:** Chunsheng Fang, Liyuan Wang, Zhuoqiong Li, Ju Wang

**Affiliations:** College of New Energy and Environment, Jilin University, Changchun 130012, China; fangcs@jlu.edu.cn (C.F.); liyuanw19@163.com (L.W.); zhuoqiong21@mails.jlu.edu.cn (Z.L.)

**Keywords:** PM_2.5_, PSCF, CWT, Northeast China, regional transport

## Abstract

Northeast China is an essential industrial development base in China and the regional air quality is severely affected by PM_2.5_ pollution. In this paper, spatial autocorrelation, trajectory clustering, hotspot analysis, PSCF and CWT analysis are used to explore the spatial pollution characteristics of PM_2.5_ and determine the atmospheric regional transmission pattern for 40 cities in Northeast China from 2016 to 2020. Analysis of PM_2.5_ concentration characteristics in the northeast indicates that the annual average value and total exceedance days of PM_2.5_ concentration in Northeast China showed a U-shaped change, with the lowest annual average PM_2.5_ concentration (31 μg/m^3^) in 2018, decreasing by 12.1% year-on-year, and the hourly PM_2.5_ concentration exploding during the epidemic lockdown period in 2020. A stable PM_2.5_ pollution band emerges spatially from the southwest to Northeast China. Spatially, the PM_2.5_ in Northeast China has a high degree of autocorrelation and a south-hot–north-cool characteristic, with all hotspots concentrated in the most polluted Liaoning province, which exhibits the H–H cluster pattern and hotspot per year. Analysis of the air mass trajectories, potential source contributions and concentration weight trajectories in Northeast China indicates that more than 74% of the air mass trajectories were transmitted to each other between the three heavily polluted cities, with the highest mean value of PM_2.5_ pollution trajectories reaching 222.4 μg/m^3^, and the contribution of daily average PM_2.5_ concentrations exceeding 60 μg/m^3^ within Northeast China. Pollution of PM_2.5_ throughout the Northeast is mainly influenced by short-range intra-regional transport_,_ with long-range transport between regions also being an essential factor; organized integration is the only fundamental solution to air pollution.

## 1. Introduction

Atmospheric PM_2.5_ pollution is of great importance to the international community because of its harmfulness, extensive coverage and being difficult to manage. The World Health Organization has confirmed that PM_2.5_ is the most harmful to human health and that it can cause cancer [1], as well as respiratory and cardiopulmonary diseases [2], increasing the morbidity and mortality of many diseases [3]. As the world’s largest developing country and a major manufacturing country, PM_2.5_ pollution in China has always been severe [4,5,6]. In 2013, China’s State Council issued the “Action Plan for Prevention and Control of Air Pollution”, with the expectation to reduce PM_2.5_ pollution in five years. Compared with 2015, the number of cities with sub-standard PM_2.5_ concentrations and the ratio of heavily polluted days dropped by more than 18% and 25% separately in 2020, while the ratio of good air quality days in prefecture-level and above cities has reached 80%.

Heavy pollution events are caused by a combination of high-intensity emissions and unfavorable meteorological conditions; the mobility of the atmospheric environment determines the regional transport characteristics of atmospheric pollutants, which can be transported from more polluted areas to downstream cities under the effect of meteorological factors, forming regional pollution, and becoming one of the important factors affecting regional air quality [7,8]. Spatial models can be used to study the dispersion of pollutants in landfills [9,10], stacks [11,12], wildfires [13,14] and urban areas [15], and it is important to apply spatial models to study PM_2.5_. However, previous studies on regional pollution have mainly focused on developed regions in China, such as Beijing–Tianjin–Hebei [16,17], Yangtze River Delta [18,19] and Pearl River Delta regions [20,21]. Regional composite pollution has currently become a characteristic of air pollution in China.

Research has shown that the degree of pollution in Northeast China could not be underestimated, especially in winter and spring [22], which is mainly caused by the increase in emissions from coal-fired power plants due to the burning of agricultural straw and the central heating from October to April of the following year [23]. The periphery of Northeast China is the high mountains and hills of the Greater Khingan, the Lesser Khingan and Changbai Mountain, the central part is the vast Northeast China Plain (Songnen Plain, Liaohe Plain, Sanjiang Plain). The special geographical environment causes strong atmospheric linkage in Northeast China; indeed, it is very feasible to conduct research on the whole for regional pollution identification and pollution transmission. In recent years, many large cities in Northeast China have seen high value areas of PM_2.5_ pollution, and the special geographical environment of Northeast China has caused strong atmospheric linkage effects and exhibited strong regional characteristics. In this context, it is important to identify potential sources of fine particulate matter pollution, to fundamentally solve the regional pollution problem of fine particulate matter in Northeast China, and to determine the regional transport pathways for the scientific prevention and control of fine particulate matter pollution.

## 2. Materials and Methods

### 2.1. Study Area and Data Processing

**Figure 1:**
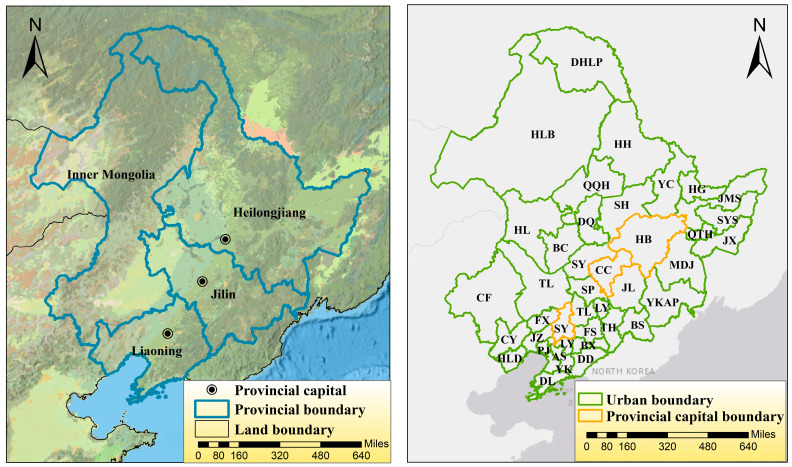
Geographical location and overview of the study area.

Administratively comprising the provinces of Liaoning, Jilin and Heilongjiang and the four eastern leagues of Inner Mongolia, Northeast China is rich in natural resources and well-developed agriculturally, making it an important grain-producing region in China. The industry is the backbone of the national economy and main source of fiscal revenue in Northeast China [24]. The geographical location of the study area is shown in Figure 1.

This study used PM_2.5_ hourly concentration data from a total of 177 air quality monitoring stations in 40 cities in Northeast China from 2016 to 2020 [25]. The full data analysis of PM_2.5_ is based on the National Ambient Air Quality Standard (NAAQS, GB 3095-2012). If the hourly value and annual mean value of PM_2.5_ exceed 75 and 35 μg/m^3^, it shall be regarded as exceeding the standard. Data were excluded including missing hourly PM_2.5_ concentration values from the original monitoring station, daily average PM_2.5_ concentration values with less than 20 h of valid data per day and monthly average PM_2.5_ concentration values with less than 27 days of valid data per month (25 days in February). The effective rate of average daily PM_2.5_ in the study area was 96.3% and 90.3% for monthly average values. The fire point data used in this study were obtained from the near real-time surface high temperature anomaly dataset SatSee-Fire published by the Institute of Remote Sensing and Digital Earth of the Chinese Academy of Sciences (CAS). The spatial vector data and arable land patches of county administrative regions in China were derived from the Resource and Environment Science Data Center (RESDC) of the CAS (https://www.resdc.cn/) (accessed on 16 July 2021), where the arable land vector patches were extracted from the 2015 remote sensing monitoring data of the current land use in China.

### 2.2. Spatial Autocorrelation and Hot Spot Analysis

Spatial autocorrelation refers to the dependence of geographical things or attributes on spatial location, and the closer the spatial location the stronger the correlation [26]. This study uses global and local Moran′s I to characterize the spatial autocorrelation of PM_2.5_ concentrations in Northeast China. The positive and negative I values indicate a positively or negatively correlated spatial distribution of pollutant concentrations. The magnitude indicates that similar concentration values (high or low values) tend to be spatially clustered or dispersed. High-value adjacent elements clustered near high-value elements are called high-high (H–H); otherwise, low-low (L–L); the adjacent elements clustered near the high-value elements with low values are called high–low (H–L); otherwise, low-high (L–H), and I = 0 means the spatial distribution of concentration is random. The specific calculation method of I value is shown in Equation (1):

(1) $$I=\frac{n\sum_{i=1}^{n}\sum_{j=1}^{n}\omega_{\mathrm{ij}}\left(X_{i}-\;\bar{X}\right)\left(X_{j}-\;\bar{X}\right)}{\sum_{i=1}^{n}\sum_{j=1}^{n}\omega_{\mathrm{ij}}\sum_{i=1}^{n}{\left(X_{i}-\;\bar{X}\right)}^{2}}$$

In Equation (1), *x*_i_ and *x*_j_ are the fine particulate matter concentration values of cities i and j in the study area, respectively, ω_ij_ is the spatial weight matrix, n is the number of cities in the study area, and $\bar{X}$ is the mean value of fine particulate matter concentration in the study area. The ω_ij_ was calculated based on whether two cities in the study area are adjacent to each other. If two cities are adjacent, the number “1” is defined, and if they are not adjacent, the number “0” is defined and so on, forming a matrix of spatial weights for all cities in the region.

After using the global Moran′s I to reflect the clustering or dispersion dynamics of the whole study area, we analyzed the clustering or dispersion effects of fine particle concentrations in local areas by calculating the $G_{i}^{*}$ values of the Getis–Ord index, and detects the specific locations of hot and cold spot areas to reflect the specific situation of the local area [27,28]. The calculation method is shown in Equation (2):

(2) $$G_{i}^{*}\left(d\right)=\frac{\sum_{j=1}^{n}\omega_{\mathrm{ij}}X_{i}-\;\bar{X}\sum_{j=1}^{n}\omega_{\mathrm{ij}}}{S\sqrt{\frac{\left|n\sum_{j=1}^{n}\omega_{\mathrm{ij}}{}^{2}-{\left(\sum_{j=1}^{n}\omega_{\mathrm{ij}}\right)}^{2}\right|}{n-1}}}$$

In Equation (2), *x*_j_ is the concentration of fine particles in city j; ω_ij_ is the spatial weight matrix; S is the standard deviation; $\bar{X}$ is the mean value of fine particles concentration in the study area, and n is the number of cities. The positive or negative $G_{i}^{*}$ value indicates that the area is a concentration zone of high (hot spot) or low (cold spot) values.

### 2.3. Backward Trajectory Model

#### 2.3.1. Cluster Analysis

The hybrid single particle Lagrangian integrated irajectory (HYSPLIT) model [29] developed by the National Oceanic and Atmospheric Administration (NOAA) and the Australian Bureau of Meteorology (BOM) was used to simulate 48 h backward trajectories at 500 m altitude for three polluted provincial capitals (Harbin, Changchun, and Shenyang) in Northeast China to analyze atmospheric pollutant transport [30]. In this paper, the Euclidean distance clustering algorithm in TrajStat software, which combines the HYSPLIT model and GIS technology, was used to cluster the airflow trajectories arriving at three provincial capitals in Northeast China to obtain different clustering results for the three cities [31,32].

#### 2.3.2. PSCF Analysis

This study used potential source contribution function (PSCF) analysis to locate pollution sources using backward trajectories. In order to reduce the error and make the results more accurate and practical, a weighting factor W_ij_ was introduced, the value of which depends on the relationship between the sum of the transmission times of all trajectories in a given grid and the average residence time of each grid, W_ij_ is expressed in Equation (3).

(3) $$W_{\mathrm{ij}}=\{\begin{array}{l} 1.0\;\;\;\;\;\;\;\;\;\;\;\;\;\;\;\;\;n_{\mathrm{ij}}>80\\ 0.7\;\;\;\;\;\;\;\;20<n_{\mathrm{ij}}\leq 80\\ 0.42\;\;\;\;\;\;10<n_{\mathrm{ij}}\leq 20\\ 0.05\;\;\;\;\;\;\;\;0<n_{\mathrm{ij}}\leq 10 \end{array}$$

The weighting factor W_ij_ used in this study reduces the uncertainty of PSCF results and is called WPSCF [33]. The grid resolution of PSCF was set to 0.5° × 0.5° [34]. The PM_2.5_ limit value used in the study is 75 µg/m^3^, which is the limit value of the secondary standard of the National Ambient Air Quality Standard (NAAQS) established by the Ministry of Ecology and Environment of China.

#### 2.3.3. CWT Analysis

The concentration-weighted trajectory (CWT) method can quantify the concentration contribution level of external transport by obtaining the average value of the concentration of samples corresponding to all trajectories passing through a single grid during the study period [35]. The CWT analysis method can obtain the difference in the pollution level of contaminated trajectories by calculating the weighted degree. In the CWT analysis method, each grid point is assigned a degree of weight [36]. By introducing the same numerical correction from W_ij_ in the PSCF method, the weighted average concentration value (WCWT value) can be used to distinguish the source intensity of potential sources. A higher WCWT value in the grid indicates that the air mass passing through the grid results in a high receiving point concentration, and the area corresponding to this grid can be considered as a potential area of high concentration contribution to the external transport of pollutants from the receiving area.

## 3. Results

### 3.1. Identification and Analysis of PM_2.5_ Pollution Characteristics in Northeast China from 2016–2020

#### 3.1.1. Analysis of PM_2.5_ Emission Reduction Effects in Northeast China

**Figure 2:**
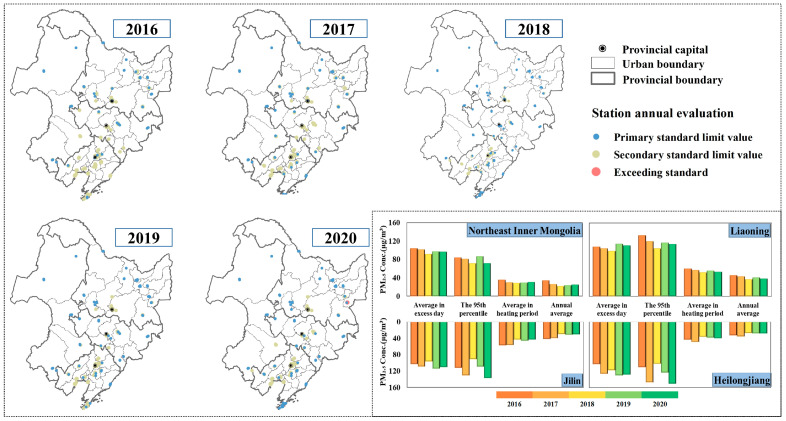
Annual average PM_2.5_ concentration compliance of air quality monitoring stations in Northeast China from 2016 to 2020. The four combined PM_2.5_ pollution indicators for each of the four regions (Northeast Inner Mongolia, Liaoning, Jilin and Heilongjiang) are shown in the bar chart.

**Figure 3:**
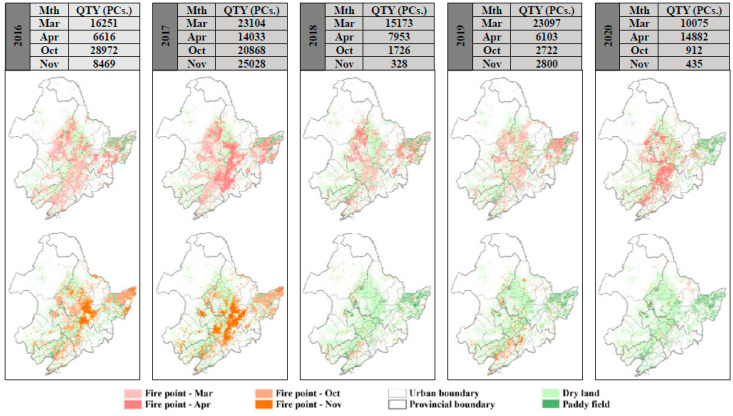
Statistical quantities (QTY) and distribution of fire point data in Northeast China in March, April, October and November 2016–2020.

Benefiting from the country’s determination to reduce pollutant emissions, China’s key regions have seen significant improvements in pollution and air quality, with numerous studies reporting that 2018 was the most effective year for combating PM_2.5_ pollution in China [37]; the annual average PM_2.5_ concentration reached the lowest value in recent years (31 μg/m^3^), decreasing by 12.1% year-on-year, and the maximum number of exceedance days was 61 days in Jinzhou, totaling 797 days in all 40 cities (Table S1 in Supplementary Materials), of which Liaoning Province accounted for 55%. However, Figure 2 shows that the average annual PM_2.5_ concentration and the total number of polluted days in Northeast China showed a U-shaped variation in the previous five years, with a slight rebound after 2018, and the total number of exceedance days increasing 30% in 2020, with an annual average PM_2.5_ concentration of 32 μg/m^3^. Excellent weather conditions are due to China’s initiatives to transform, remediate, eliminate and clean up the industry in accordance with its industrial structure [38]. Initiatives in Northeast China have focused on managing open biomass burning and coal combustion emissions during the heating period. Figure 3 shows the fire point data set for March, April, October and November in Northeast China yearly, which has a suitable identification of abnormally high-temperature points, including straw and burning fire points [39]. The result shows that the number of fire points increased significantly to a peak in 2017, with these four months accounting for approximately 83% of the year, of which 91% are concentrated on the subsurface of farmland.

The stringent treatment of local governments in Northeast China has sharply reduced straw burning in the region. By 2020, the number of fires across the region had been reduced by 68% from their peak, with a 97% reduction in the two winter months. It is fair to say that reasonable control and organized straw burning under favorable diffusion conditions have effectively controlled pollution. The statistics in Figure 2 show that the pollution in winter in the Northeast China has been controlled, with an average PM_2.5_ concentration of 44.6 μg/m^3^ during the heating period. Meanwhile, the PM_2.5_ pollution has started to rebound in several regions in Northeast China after 2018, with the 95% quantile being the most pronounced, increasing at a rate of 1–2% per year.

Although emission reduction has been the dominant factor in China’s air quality improvement in recent years, and studies in previous years have tended to attenuate the influence of meteorological factors, it is now generally accepted that the implementation of regional joint prevention and control of air pollution has been an effective means of addressing air pollution prevention [40,41]. As shown in Figure 2, the PM_2.5_ pollution assessment of 177 state-controlled monitoring stations showed, with the exception of individual stations (Jixi Water Company and Baijiu Factory Station, Jixi, China) where PM_2.5_ concentrations exceeded the national ambient air quality standards by approximately 15% in 2020, all other stations meet the standards, of which 42% of the stations met the primary standard (35 μg/m^3^). Higher PM_2.5_ concentrations were concentrated in three provincial capitals and surrounding cities, of which Jilin Province is sandwiched in the middle and has slightly better indicators than the other two provinces caused by the transmission from the highly polluted regions [42]. Previous studies concerning the perennial air mass trajectory movement pattern prove have shown that more than 60% of the polluted air masses come from Inner Mongolia in the north direction of Northeast China [43]. In contrast, Liaoning Province borders Bohai Bay and variable weather systems following the reduction of local emissions have resulted in the region being more affected by meteorological transport. Strong northerly winds in winter and spring rapidly import polluted air masses from the Beijing–Tianjin–Hebei region upstream into Liaoning Province. Rapids transport and continuous water vapor accumulation due to the difference in pressure between land and incoming sea in summer favors the transport and stagnation of pollutants over Liaoning Province [44].

#### 3.1.2. Spatial and Temporal Characteristics of PM_2.5_ Pollution in Northeast China

**Figure 4:**
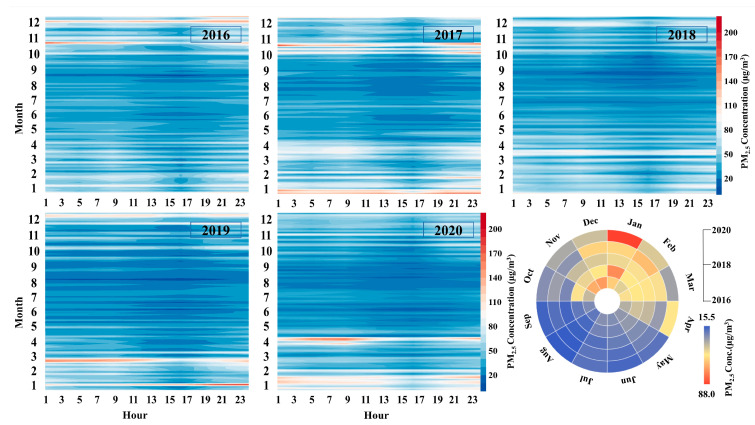
Time series of PM_2.5_ concentration in Northeast China from 2016 to 2020. The stacked color graphs present the hourly mapping of PM_2.5_ concentrations and the pie charts show the variation in monthly average PM_2.5_ values.

The yearly hour PM_2.5_ pollutant concentration distribution throughout Northeast China (Figure 4) and each city (Figures S1–S5 in Supplementary Materials) shows that the beginning and end of each year are the most polluted periods for PM_2.5_ pollution. In a side-by-side comparison, 2018 remains the least polluted year, with maximum hourly PM_2.5_ concentrations around 50% lower than 2016. 2020 contributes the highest average January concentrations in the Northeast China in those years at 88μg/m^3^. As a result of the epidemic lockdown, the country was completely closed in February and March in 2020. When operations resumed in various industries in early April, severe PM_2.5_ pollution broke out for 7 days in Northeast China with the most polluted day reaching a daily average of 155.9 μg/m^3^, which peaked at 807 μg/m^3^ on 13 April in Qiqihar, and hourly PM_2.5_ concentrations in individual cities even exceeding 1000 μg/m^3^ on 18 April. This outbreak was still associated with straw burning, which is commonly carried out on a large scale in the region before spring plowing. At the same time, unfavorable meteorological conditions of rising relative humidity and ground-level winds of less than 2 m/s in Northeast China occurred during this period, resulting in air quality maintaining heavy pollution levels for a long time [45]. In previous years, PM_2.5_ pollution levels in Northeast China always spiked to a peak at the beginning of the heating period and decreased until the end of the following spring, which was higher in the end of each year from October to December than that of the current and following year from January to April. Nevertheless, it becomes the case that PM_2.5_ pollution is higher in the first half of the year than in the second half since 2017, a situation that is more clearly reflected in the results of the fan chart in Figure 4, which shows the average values each month for the 5 years. In general, the highest PM_2.5_ pollution period is from October to April, As the temperature rises, the boundary layer rises, turbulent vertical exchange is enhanced and PM_2.5_ diffusion is enhanced [46], a rare low-pollution moment of the day for PM_2.5_ is between 15:00 and 17:00. Later in the evening, as the temperature decreases, the boundary layer height decreases, the turbulent vertical exchange weakens and the PM_2.5_ concentration increases.

**Figure 5:**
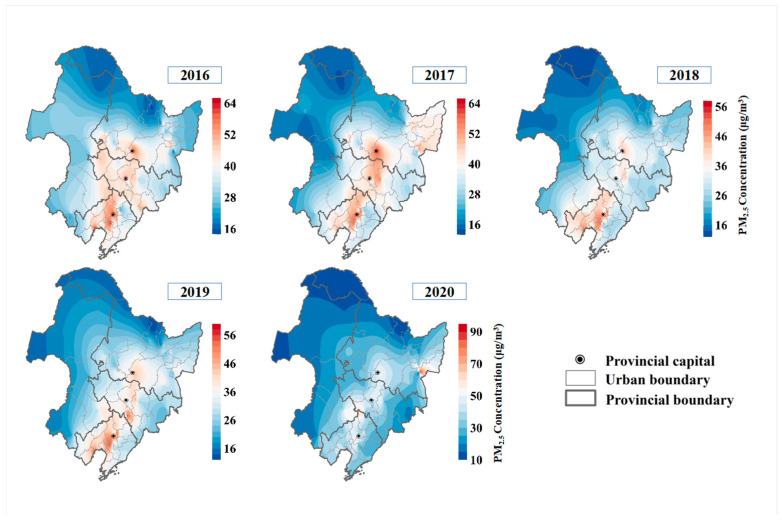
Spatial distribution of PM_2.5_ concentration in Northeast China from 2016 to 2020.

The spatial distribution of annual average PM_2.5_ concentrations in Northeast China is shown in Figure 5. It is evident that the ambient atmosphere in the Northeast China has experienced a contiguous massive patch of PM_2.5_ pollution yearly and monthly (Figure 1, Figure 2, Figure 3, Figure 4 and Figure 5 attached) for the past five years. The heavily polluted areas are concentrated in and around the three provincial capitals, with the polluted areas extending in a band from the Southwest to Northeast China. In 2020, there is a center of high concentration appears in Jixi City, Heilongjiang Province, where the concentration is more than 70% higher than the surrounding area. The analysis of PM_2.5_ spatial distribution proves that a linked atmospheric pollution belt has formed in Northeast China and regional transport has become a significant factor in local pollution sources. The influence of regional transmission accounts for more than 70% in the heavy pollution. Northeast China must tighten its grip on joint prevention and control while addressing local pollution in order to achieve PM_2.5_ concentrations at new lows, through improvement of air quality and control

### 3.2. Characteristics of Spatial Agglomeration Patterns in Northeast China from 2016–2020

**Figure 6:**
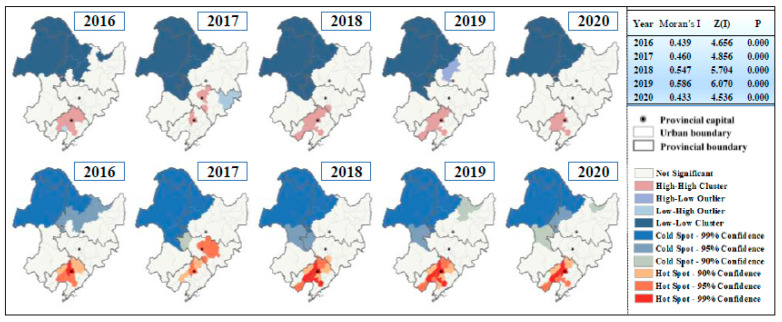
Results of spatial autocorrelation analysis and hot spot analysis of PM_2.5_ concentration in Northeast China from 2016 to 2020. The detailed calculations are shown in the table on the right.

The results of the global spatial autocorrelation analysis of PM_2.5_ concentrations indicated that the Moran′s I > 0 for PM_2.5_ concentrations in Northeast China from 2016 to 2020, and all passed the significance test of 99%, with the Z scores were well above the critical value of 2.58 [47]. Therefore, the distribution of PM_2.5_ concentrations in Northeast China has a significant positive spatial autocorrelation, and cities with similar concentrations tend to be clustered, which has become more assertive in recent years. The local spatial autocorrelation analysis in Figure 6 reveals that the northwestern part of Northeast China is always in the low–low (L-L) clustering category, and the high–high (H-H) clustering phenomenon is always found in the pollution belt of Liaoning Province in the northeastward extension of Liaodong Bay. Around these two kinds of clustering areas, low–high (L–H) and high–low (H–L) clustering emerged in 2017 and 2019 respectively, indicating that cities are increasingly influencing each other. With the pollution situation of a single city being influenced by the surroundings evolving into a convergence pattern, the PM_2.5_ clustering conditions of individual cities in Northeast China are becoming more consistent from 2018 to 2020.

Using Getis–Ord $G_{i}^{*}$ to identify clustering areas with positive spatial autocorrelation, the results show that PM_2.5_ concentrations in Northeast China in the past five years have a clustering characteristic of being hot in the south and cold in the north. The hotspot cities that passed the significance test for annual average PM_2.5_ concentrations were mainly distributed in Liaoning Province, indicating that these regions are indeed the high PM_2.5_ pollution areas in Northeast China. The cold spot cities are concentrated in the western part of Heilongjiang Province and eastern part of Inner Mongolia, with excellent ambient air quality in Northeast China. The hotspot areas in Liaoning Province have gradually shrunk in the last three years, although the distribution of cold spot cities is stable, besides spread out tendency for PM_2.5_ concentration due to pollution from surrounding cities.

### 3.3. Characteristics of the Source Trajectory of Air Masses in Northeast China in 2020

**Figure 7:**
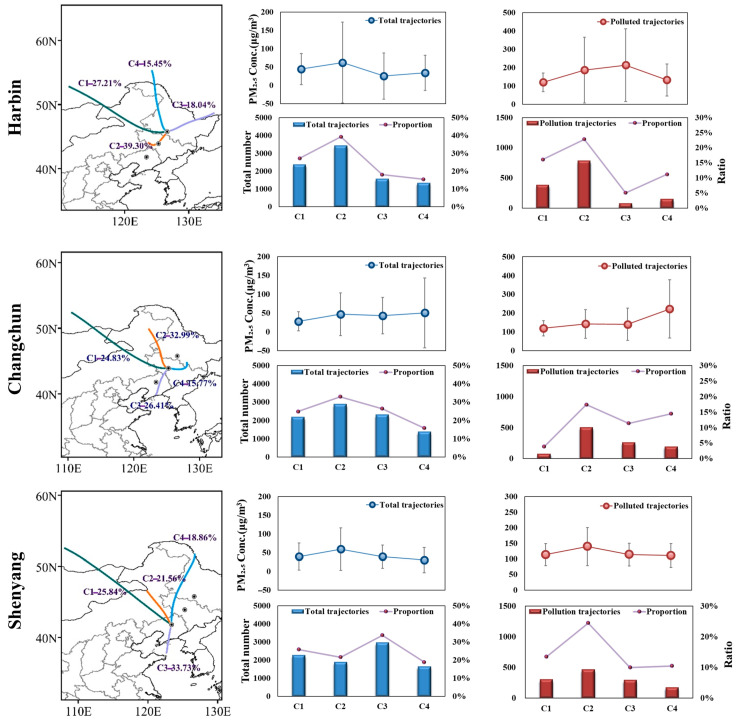
Backward trajectory analysis and statistical results of the total number of cleaning and pollution trajectories in Harbin, Changchun and Shenyang in 2020.

The above analysis indicates that the regional transport characteristics of Northeast China are already formed. It is necessary to reveal its pollution transport patterns. Consequently, three heavily polluted cities in Northeast China (Harbin, Changchun and Shenyang), which are also provincial capitals, were selected for more focused discussion in 2020. We apply the HYSPLIT model to determine the transport paths of atmospheric pollutants and the results are shown in Figure 7 [48,49]. All trajectories were clustered into four entries named cluster C1 to C4. Cluster C2 carries the most significant number of trajectories in Harbin City and accounting for 39.3% of the total 8784 trajectories in 366 days multiplied by 24 h, which also wrapped the most polluting trajectories totaling 787, whose average PM_2.5_ pollution concentration reached 186.6 μg/m^3^, nearly two times higher than the total average concentration. Cluster C2 originates from the southwest and the western part of Jilin Province, and then blows straightly through Changchun, with a shorter path and moves more slowly, carrying pollutants from Jilin Province to Heilongjiang Province. The trajectory clustering results for Changchun show that about 58% of trajectories come from the northwest, with the most significant proportion of the Cluster C2 (33%) coming from Northeast Inner Mongolia. However, the Cluster C4 comes from southern Heilongjiang Province with a minor proportion (15.8%) and contributes more polluting trajectories with an average PM_2.5_ concentration of 222.4 μg/m^3^. The Cluster C3 in Shenyang accounted for the largest proportion and wrapped the least polluted trajectory (9.9%), but the average PM_2.5_ of the polluted trajectory was as high as 113.8μg/m^3^, which is strongly related to the polluted air mass from the intersection of the Yellow Sea and Bohai Sea.

Generally, the majority of the air mass paths from these three cities transmit within the study region, transporting and influencing local air quality with each other. However, 26% of the trajectories originate from Mongolia, which is outside the study area. Numerous studies have demonstrated that long-range transport from Mongolia has been the largest source of air pollution affecting Jilin Province for many years [50]. In this paper, the analysis within Northeast China proves that short-range regional transport instead transmits more air pollutants than long-range cross-regional transport, which indicates that delineating local areas for joint pollution control is an essential option.

### 3.4. Analysis of Potential Source Contributions and Concentration Weighting Trajectories in Northeast China 2020

**Figure 8:**
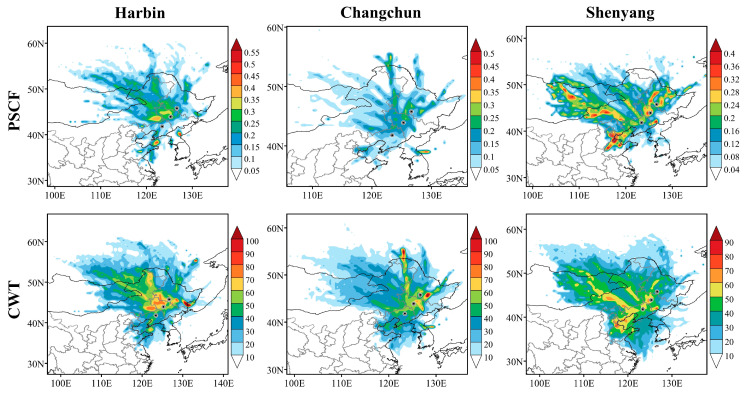
PSCF and CWT analysis results of Harbin, Changchun and Shenyang in 2020.

The cluster analysis of the trajectories has clearly determined the transmit direction of trajectories over Northeast China, the proportion and the concentration of the polluted trajectories. In order to specifically determine the relative contribution of potential source areas of the study area, we conducted PSCF analysis of PM_2.5_ in Northeast China throughout 2020, while the CWT method was used to analyze the concentration weight trajectories, which better reflects the specific contribution concentration of the pollution grid [51,52]. For the analysis of the results shown in Figure 8, it was found that the main potential source areas (PSCF > 0.4) in Harbin were distributed at the junction of the Yellow Sea and the Bohai Sea with the eastern part of Korea. The distribution of moderate potential source areas (0.3 < PSCF < 0.4) in Harbin is concentrated in the northwestern part of Jilin Province and the border between Inner Mongolia and Liaoning Province, the high-value areas of the PM_2.5_ concentration weighting trajectory are also distributed in this range with a daily average PM_2.5_ concentration contribution of 70–80 μg/m^3^. CWT analysis reveals the important influence of short-range regional transport on air pollution, Jixi City, located directly east of Heilongjiang Province, contributes more than 100 μg/m^3^ to the daily average PM_2.5_ concentrations in Harbin.

Most of the PSCF (PM_2.5_) values in Changchun are below 0.3, and the light pollution grids are mainly distributed in the high pollution belt in the Northeast China, contributing 60–70 μg/m^3^ to the daily average PM_2.5_ concentration. A small proportion of the medium to heavy pollution grids are scattered in the east of Korea, contributing around 90–100 μg/m^3^ to the PM_2.5_ concentration in Changchun. Shenyang has the lowest overall contribution factor values for PM_2.5_ of the potential source area and the pollution grids are very loosely distributed throughout Northeast and Eastern Inner Mongolia. The PM_2.5_ concentration weighting trajectory analysis, on the other hand, better presents the extent to which the study area contributes to the PM_2.5_ concentration in Shenyang. The high-value areas of the concentration weight trajectory in Shenyang are mainly located in the pollution belt of Northeast China, and some of the high-value areas are from the heavily polluted Inner Mongolia region. This shows that the influence of regional transmission caused by atmospheric flow is powerful between regions and regions themselves. Therefore, dividing the whole region and organized linkage and integration is the fundamental solution to air pollution.

## 4. Discussion

The results of identifying the characteristics of PM_2.5_ concentration variations in Northeast China from 2016 to 2020 show that the PM_2.5_ emission reduction effect of straw burning and coal-fired heating is significant, with an average PM_2.5_ concentration of 44.6 μg/m^3^ in spring and winter. The average annual PM_2.5_ concentration and the total number of polluted days in Northeast China from 2016 to 2020 show a U-shaped change, with about 42% of monitoring stations meeting a primary air quality standard. Northeast China had the best air quality in 2018. The annual average PM_2.5_ concentration reached a minimum of 31μg/m^3^ and decreased 12.1% year-on-year, after which the indicators rebounded slightly, with the annual average PM_2.5_ concentration increasing at an annual rate of 1–2%. An outbreak of PM_2.5_ pollution in January and April in 2020 due to the epidemic lockdown, with the highest daily average value across the Northeast China reached 155.9 μg/m^3^. In contrast to this phenomenon in northeast China, studies in the Beijing–Tianjin–Hebei (BTH) region and the North China Plain (NCP) in China also found an increase in PM_2.5_ concentrations during the lockdown, which was related to anthropogenic sources such as heating and fireworks emissions and straw burning during the pandemic [53,54,55]. In other countries such as the United States and India, decreases in PM_2.5_ concentrations were observed to varying degrees [56,57]. Spatially, the pollution in Northeast China has become contiguous, with spatial interpolation results indicating the emergence of a pollution belt in Northeast China extending from the Southwest to Northeast China, landing in Liaoning Province from Liaodong Bay, connecting the three provincial capitals and running southwest towards Heilongjiang Province. The whole Northeast China shows a high degree of spatial autocorrelation, and exhibits the clustering characteristics of south-hot-north-cool. Coving all hotspot areas, Liaoning Province is the most polluted region in terms of PM_2.5_ and shows H-H clustering.

For the three heavily polluted cities in 2020, the analysis of the results found that more than 74% of the backward trajectories were transported between these three provincial capitals. Forty percent of the air masses in Harbin originate in the western part of Jilin Province and carry the most pollution backward trajectories, with average PM_2.5_ concentrations reaching 186.6 μg/m^3^. Most of the backward trajectories transport through Changchun are from Inner Mongolia, but the most pollution trajectories come from southern Heilongjiang province, with average PM_2.5_ concentrations in these polluting air masses reaching 222.4 μg/m^3^. The pollution in Shenyang is influenced by multiple factors, including air masses from Inner Mongolia and the pollution belts in the study area, while air masses from the interface between the Yellow Sea and the Bohai Sea also contribute a large proportion of PM_2.5_ pollution. Similar results were obtained in the studies for the central cities of Liaoning Province as well as Shenyang City, suggesting some commonality in the results of backward trajectory analysis in Northeast China [58,59].

The results of analyzing the potential source contribution and concentration weight trajectories of the three heavily polluted cities in 2020 show that the distribution of potential source areas in Harbin City is relatively concentrated, with the main potential source areas distributed at the junction of the Yellow Sea and the Bohai Sea with the eastern part of North Korea. The moderate potential source areas are distributed in the northwestern part of Jilin Province and the junction of Inner Mongolia and Liaoning Province, consistent with the distribution range of the concentration weight trajectories, which contribute daily average PM_2.5_ concentrations over 70 μg/m^3^. There are no major potential source areas of PM_2.5_ pollution in Changchun, and the light pollution grids are mainly distributed in the high pollution belt in Northeast China, with a contribution of 60–70 μg/m^3^ of daily average PM_2.5_ concentration, and a part of the grids with a contribution of more than 90 μg/m^3^ scattered in the east side of Korea. The PM_2.5_ pollution grid in Shenyang is loosely distributed across Northeast China and Eastern Inner Mongolia. Most of the high PM_2.5_ concentration weighting trajectory areas are located in the northeast China pollution belt, with a small proportion coming from the heavily polluted Inner Mongolia region. In the study for Beijing, some similarity was found between the potential source areas and high concentration contribution areas in Beijing and the results for Northeast China; higher results values were also found in Shandong, Henan and Hebei [60]. In the study for the southern cities, the main potential source areas and high contribution areas were concentrated in the Yangtze River Delta region [61].

## 5. Conclusions

Throughout China, there is an apparent regional pollution pattern that has emerged. Meanwhile, numerous large cities in Northeast China are considered high PM_2.5_ pollution areas and have shown solid regional characteristics for pollution. In this context, a scientific and detailed anatomizing of the sources of PM_2.5_ is essential to resolve the regional pollution in the Northeast China fundamentally and to identify regional transmission pathways for the scientific prevention and control of PM_2.5_ pollution. It is of tremendous research significance to explore the spatial pollution characteristics of PM_2.5_ in Northeast China and determine its atmospheric regional transmission pattern, providing a basis for the study of linked atmospheric pollution and prevention and control measures. Straw burning has been a major contributor to the occurrence of heavy pollution in the northeast from the beginning to end. Local governments in the northeast have invested heavily in various monitoring, guidance, and accountability approaches and have largely contributed to tackling the straw-burning problem. Importantly, this study clarifies that PM_2.5_ pollution in the northeast has spatial clustering characteristics and that Liaoning Province is the most heavily polluted hot spot for PM_2.5_ pollution.

The internal pollution trajectories in Northeast China transmit an exceptionally high proportion of pollution; the main potential source areas of pollution are spread across the province and parts of the surrounding countries. This paper also identifies a high pollution belt in the Northeast, which has the most severe PM_2.5_ pollution and most robust spatial clustering, seriously affecting the air quality of the surrounding region. The findings of this paper are important for research on the regional linkage of air pollution transmission corridors in the northeast, which is consistent with the strategic trend of China’s future air quality objectives, which is the sub-regional joint prevention and control of air pollution.

## Data Availability

Publicly available datasets were analyzed in this study. This data can be found here: [http://www.cnemc.cn/] (accessed on 24 November 2021).

## Supplementary Materials

The following are available online at https://www.mdpi.com/article/10.3390/ijerph182312483/s1, Figure S1: Monthly spatial trends in PM_2.5_ in the Northeast China in 2016, Figure S2: Monthly spatial trends in PM_2.5_ in the Northeast China in 2017, Figure S3: Monthly spatial trends in PM_2.5_ in the Northeast China in 2018, Figure S4: Monthly spatial trends in PM_2.5_ in the Northeast China in 2019, Figure S5: Monthly spatial trends in PM_2.5_ in the Northeast China in 2020, Table S1: Total excessive days of all cities in Northeast China from 2016 to 2020.
