# Supplementary material for: Spatial Characteristics and Regional Transmission Analysis of PM2.5 Pollution in Northeast China, 2016–2020
Source: Int J Environ Res Public Health. 2021 Nov 26;18(23):12483. doi: 10.3390/ijerph182312483 (PMC8657314; doi:10.3390/ijerph182312483)
Supplement: Supplementary file 1 [file ijerph-18-12483-s001.zip › ijerph-1440152-supplementary.pdf]

Table S1. Total excessive days of all cities in Northeast China from 2016 to 2020

Province	City	Abbreviation	Summary of excessive days				
			2016	2017	2018	2019	2020
Northeast Inner Mongolia	Chifeng	CF	22	21	12	6	5
	Tongliao	TL	30	28	16	27	18
	Hulunbuir	HLB	4	3	0	1	1
	Hinggan League	HL	12	5	0	8	8
Liaoning Province	Shenyang	SY	75	64	33	39	41
	Dalian	DL	27	28	16	33	21
	Anshan	AS	52	59	19	38	38
	Fushun	FS	37	62	46	50	54
	Benxi	BX	39	36	11	35	29
	Dandong	DD	34	26	9	18	15
	Jinzhou	JZ	71	58	61	50	55
	Yingkou	YK	42	44	33	48	38
	Fuxin	FX	36	38	33	31	30
	Liaoyang	LY	45	64	29	47	42
	Panjin	PJ	27	42	29	40	31
	Tieling	TL	55	72	34	40	44
	Chaoyang	CY	31	38	38	30	30
	Huludao	HLD	44	50	44	50	38
Jilin Province	Changchun	CC	53	62	19	37	46
	Jilin	JL	42	69	24	35	45
	Siping	SP	45	59	33	25	32
	Liaoyuan	LY	59	57	20	29	48
	Tonghua	TH	42	20	7	14	11
	Baishan	BS	51	42	4	6	3
	Songyuan	SY	25	34	17	26	28
	Baicheng	BC	46	21	10	24	11
	Yanbian Korean Autonomous Prefecture	YKAP	20	18	13	10	3
Heilongjiang Province	Harbin	HB	73	76	38	44	53
	Qiqihar	QQH	31	29	14	20	23
	Jixi	JX	13	38	23	18	11
	Hegang	HG	16	26	3	4	4
	Shuangyashan	SYS	20	29	14	12	17
	Daqing	DQ	31	34	13	19	28
	Yichun	YC	1	8	0	5	5
	Jiamusi	JMS	18	36	21	18	22
	Qitaihe	QTH	27	47	13	23	13
	Mudanjiang	MDJ	33	33	18	22	27
	Heihe	HH	2	11	1	0	6
	Suihua	SH	23	43	28	32	46
	Da Hinggan Ling Prefecture	DHLP	13	4	1	2	0
Northeastern China			1367	1534	797	1016	1020

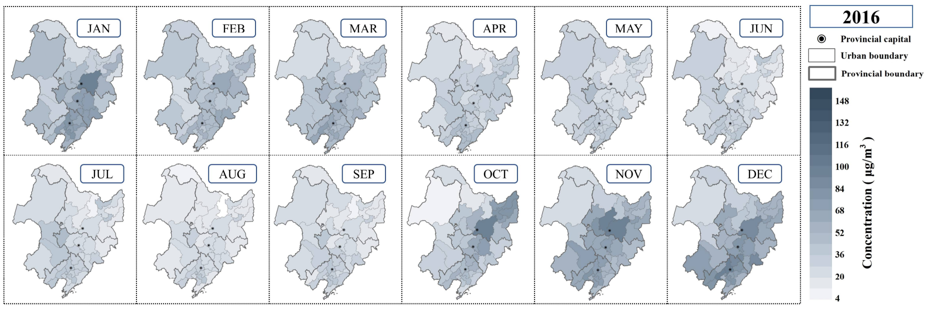

Figure S1. Monthly spatial trends in $PM_{2.5}$ in the Northeast China in 2016

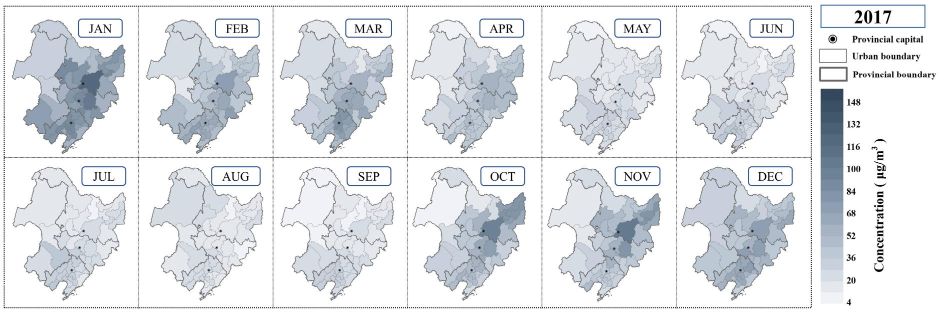

Figure S2. Monthly spatial trends in $PM_{2.5}$ in the Northeast China in 2017

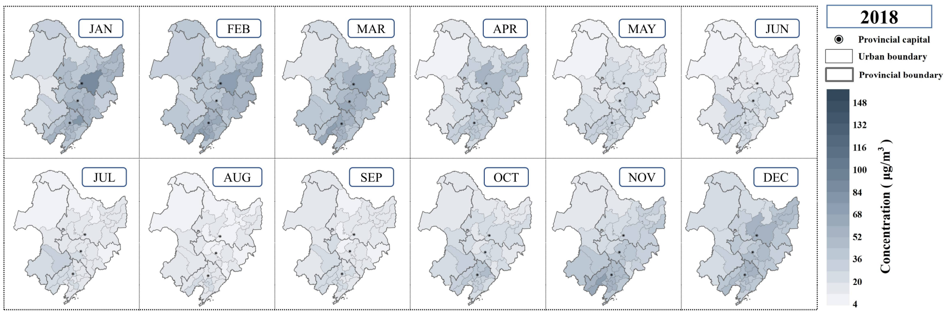

Figure S3. Monthly spatial trends in $PM_{2.5}$ in the Northeast China in 2018

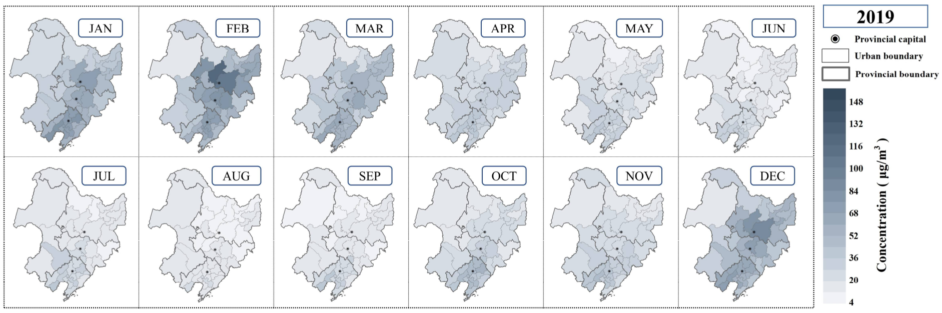

Figure S4. Monthly spatial trends in $PM_{2.5}$ in the Northeast China in 2019

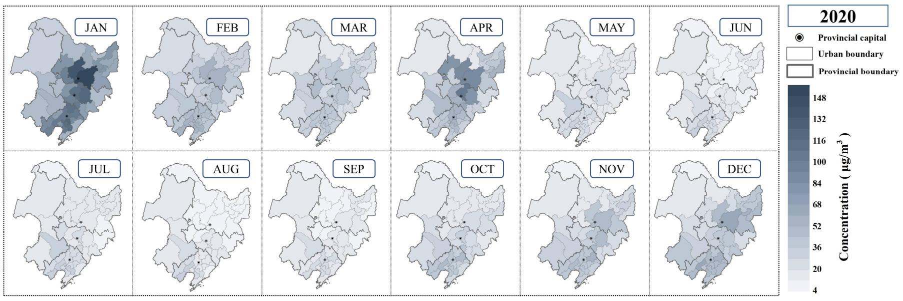

Figure S5. Monthly spatial trends in $PM_{2.5}$ in the Northeast China in 2020
